# Cycloplegic Effects on the Cylindrical Components of the Refraction

**DOI:** 10.1155/2021/8810782

**Published:** 2021-04-02

**Authors:** Athar Zareei, Milad Abdolahian, Shahram Bamdad

**Affiliations:** Poostchi Ophthalmology Research Center, Department of Ophthalmology, School of Medicine, Shiraz University of Medical Sciences, Shiraz, Iran

## Abstract

It is important to predict which astigmatic patients require separate refraction for near vision. This study compared cylindrical components changes by cyclopentolate 1% for the low and high amount of astigmatism. The right eyes of 1014 healthy individuals (307 males and 707 females) with cylindrical refractive power more than −0.5 diopter on autorefractometer were selected. Both male and female patients in the age range of 17–45 years were refracted before and after cycloplegia, using 1% cyclopentolate. All volunteers were classified into 2 subgroups including the lower astigmatism group (−2.25 to −0.50) and the higher astigmatic group (−2.50 to over). Alpines' method was used to compare the effect of cycloplegic drop on cylindrical power. The mean age in the lower astigmatism group (29.58; 95% CI: 29.18 to 29.99 years) was not significantly different from the higher astigmatic group (29.85; 95% CI: 29.07 to 30.62) and there were no significant differences in gender between these two groups (*P*=0.54). Differences between wet and dry refraction in J0 (−0.03; 95% CI:−0.06 to −0.008) and J45 (−0.03; 95% CI:−0.06 to −0.01) were significant only in the higher astigmatic group. Axis changes by the cycloplegic drop in the lower astigmatism group were 3.51 (CI: 3.22 to 3.81) and axis changes by the cycloplegic drop in the higher astigmatism group were 2.21 (CI: 1.73 to 2.49). In patients with a lower amount of astigmatism (−2.25 to −0.50), additional near subjective refraction could be done for precise determination of axis and in patients with a higher amount of astigmatism (−2.50 to over), near subjective refraction might be done for precise determination of power.

## 1. Introduction

In clinical practice, astigmatism is a common refractive condition. Although lots of investigations have been performed into the possible etiology of astigmatism, no single model or theory of the decisive development of astigmatism has been found. Despite extensive research, the exact cause of astigmatism is still not known. One potential explanation behind astigmatic development would be a hereditary etiology. The studies into genetics and astigmatism do cause controversies. Some degree of astigmatism heritability was indicated in some studies and *s* an autosomal dominant mode of inheritance has also been identified. It seems that both genetic and environmental factors play roles in astigmatic developments. Other environmental possible causes would be the tension of extraocular muscles, eyelid pressure on the cornea, and visual tasks in which astigmatism develops in response to visual cues. In a number of ethnic groups and diseases, the interaction between the cornea and the eyelids appears to be a possible reason for increased astigmatism. Studies show that the eyelids pathology and eyelid pressures during reading could also cause changes in corneal astigmatism [1]. Research into high astigmatism children shows that eyelids affect the axis and degree of corneal astigmatism [2]. Although it is clear that the eyelids can have an influence on corneal shape, there is still no evidence that conclusively shows that eyelids pressures cause corneal astigmatism.

At birth, children exhibit a high incidence of astigmatism which is corneal in origin. As children grow older, the cornea flattens with significantly reduced astigmatism. Over the age of four years, the prevalence of large amounts of astigmatism is low, with small amounts of WTR astigmatism which is found most commonly. Young adult subjects typically display small degrees of WTR astigmatism and in older adults, a shift in astigmatism occurs where ATR astigmatism becomes more prevalent. Astigmatism most commonly occurs due to the curvature of the cornea and the changes in astigmatism occurring throughout life. It also appears to be due primarily to corneal changes. The shift from with-the-rule to against-the-rule astigmatism in the cornea progresses continuously with aging. [3] The causes of this shift are unknown, though there are many hypotheses for it. Marin-Amat (43) suggested that a combination of two factors may be operative. One factor is the pressure caused by the eyelids in early life, which diminishes with progressive weakness of the orbicularis in old age. Another factor is the action of the extrinsic ocular muscles, particularly the medial rectus muscle. The action of this muscle in convergence flattens the horizontal meridian of the cornea and perhaps also makes the vertical meridian more curved, and thus may increase the with-the-rule astigmatism. This action decreases with aging. Recently, Mori Alessandro et al. investigated the relationship between the aging shift in corneal astigmatism and the action of extraocular muscles. They suggested that the progressive axial shift in corneal astigmatism with aging might be correlated with decreased action of the superior rectus muscle. However, it is difficult to conclude that the change is due to these factors, which have such individual variations. Conversely, age-related factors such as intraocular pressure are considered to produce a change in the cornea. Duke-Elder45 suggested that when an elevated intraocular pressure assumes the preponderant role in the conformation of the globe, as in glaucoma, against-the-rule astigmatism may tend to develop. However, he also indicated that this effect shown in the eyes of experimental animals is not seen regularly in the clinic. Thus, he suggested that the cause is more fundamental, involving a peculiarity of growth since the corneal curvature forms a part of a similar deformation of the anterior segment of the globe that includes the sclera. Although the possible etiology of astigmatism has been assessed, no conclusive model or theory about the development of astigmatism has not been found. [4].

There are various types of correction for astigmatism including using corrective lenses by glass prescription, contact lenses, and Orthokeratology. While using glasses, contact lenses, and orthokeratology can temporarily treat the astigmatic effects, different types of surgeries are the ways to permanently correct astigmatism. Radial keratotomy (RK) is a very popular surgical procedure to correct astigmatism in the 90s. In the following years, photorefractive keratectomy (PRK) and laser assisted in situ keratomileusis (LASIK) were more acceptable in the correction of astigmatism [5]. Laser refractive surgeries have been performed for more than 20 years in order to correct astigmatism and there have been scientific reports recording the long-term results [6].

Previous studies had shown that low levels of uncorrected astigmatism caused problems for the patient in near-work and far-work activities. [7]. Correcting astigmatism in order to reach excellent visual outcomes is necessary. [8–10]. The natural accommodative process of the eye, however, prevents accurate refractive diagnosis and prescription since the accommodative process, consciously or unconsciously, influences far-field readings of a patient. The accommodation mechanism increases the lens thickness and ultimately leads to the accommodative refractive power with a sharp near vision. [11] In order to enable accurate refractive measurements, cycloplegic drops pharmacologically are used to paralyze the ciliary muscles of the eye and as a result prevent the ocular accommodative responses [12, 13]. It is to be noted that cycloplegic drops only result in the loss of accommodation without any effects on oculomotor processes such as cyclotorsion and vergence [14]. Although Cycloplegia generally improves refractive error diagnosis, in astigmatic refractive errors, it results in an inaccurate diagnosis due to paralyzed accommodation [9, 15]. Here, we quantify the relation between cycloplegia and astigmatic change to enable a better and accurate refractive diagnosis.

Astigmatic refractive errors are conventionally reported with two values: the cylindrical lens power and the cylindrical lens axis. The cylindrical power indicates the diopter of the corrective cylindrical lens and the axis presents the degree of the angle at which the cylinder is placed. These astigmatic values are dependent on each other. For example, any change to the crystalline lens due to cycloplegia can affect cylindrical power and its axis at the same time. Power vector analysis, on the other hand, is a method that summarizes the refractive errors in a way that the reported values become independent. In power vector analysis, the refractive error is reported with three numbers: (i) *M* which is the equivalent sphere refractive error, (ii) J0 which is the Jackson cross-cylinder component with equal powers but an opposite sign at the 90 and 180 degrees, and (iii) J45 which is the Jackson cross-cylinder components with equal powers but an opposite sign at 45 and 135 degrees. It is to be noted that conventional astigmatic changes are summarized independently within J0 and J45 values. As a result, the effect of cycloplegia on astigmatic errors is studied on the values of J0 and J45. Note that cycloplegia affects accommodation and since accommodation affects near-field vision, in routine clinical practice, it has been often seen that astigmatic patients require a separate prescription for near-work activity due to visual symptoms [16, 17]. In routine examinations, the question arises as which patient needs to be prescribed a separate prescription for near-work activities. This suggests a new clinical procedure to anticipate which astigmatic patients require near-field refraction. Specifically, in this study, we classified patients according to their astigmatic power into two groups: (a) lower astigmatism group (−0.50 to −2.25) and (b) higher astigmatic group (−2.50 to over). We then studied the effect of cycloplegic drop on different astigmatic cylindrical components in these groups. Our goal is to know whether cycloplegic drop changes astigmatic values in these two groups.

## 2. Materials and Methods

At the Refractive Surgery Center, complete refractive examinations were done for 1600 individuals aged from 17 to 45 years old (29.64 ± 5.84) who wanted to perform refractive surgery. Among them, the right eyes of 1014 healthy individuals (307 males and 707 females) with autorefractometer cylindrical power more than −0.5 diopter were selected. The patients were excluded if they had any other systemic disease, previous or present eye disease or injury, amblyopia, and previous ocular surgery that would affect the refractive status. After explaining the study objective, a consent form was signed by the patients. This research followed the tenets of the Declaration of Helsinki. Eye examination was conducted by an experienced optometrist. Dry and cycloplegic autorefraction was performed, using autorefractometer (RM-8900; Topcon, Tokyo) by automatically averaging the three measurements. In order to overcome the malposition of the subject's head throughout the testing time, we tried to carefully adjust the forehead bar and chinrest for every individual. The head position was monitored carefully by keen observation during the cycloplegic or dry refraction. Cycloplegia was induced with three drops of cyclopentolate 1%, with a five-minute interval. Cycloplegia and pupil dilation were evaluated after 30 minutes for a complete cycloplegic effect. All volunteers were classified into 2 subgroups based on the cylindrical power including the lower astigmatism group (−2.25 to −0.50) and the higher astigmatic group (−2.50 to over). There were no significant differences between these two groups in age (*P*=0.377) and gender (*P*=0.543). Two different methods were used to find the effect of cycloplegia on the power and axis of astigmatism. In the first method, arithmetic changes in the power and axis of the cylinder before and after cycloplegia were analyzed and compared. In order to calculate the degree of change in the cylindrical axis, we reduced the astigmatism axis (cylindrical axis in the wet mood–cylindrical axis in the dry mood), and the absolute value was taken. If this value was below 90 degrees, it was recorded and if it was more than 90 degrees, it was subtracted from 180 degrees (180-Icylindrical axis in the wet mood–cylindrical axis in dry moodI) and recorded as cylindrical axis changes [18]. Second, the Alpins method was used to compare the effect of cycloplegic drop on cylindrical power. All refractive data (S, *C* ×*a*: *S* = spherical power, *C* = cylinder power in minus, *a* = cylinder axis) was divided into three independent components, including the *M*, J0, and J45, using the vector analysis method [19]. M parameter represented spherical equivalent power and was calculated by the following formula: *S*+ 0.50× C. J0 parameter represents the cylinder magnitude in the 90° and 180° axes and was calculated by the following formula: *J*0 = C/2cos2a. J45 parameter represented the cylinder power in 45° and 135° axes and was calculated, using the following formula: J45 = C/2sin2a. The shifts of J0 and J45 by cycloplegia were investigated at different cylindrical groups. To investigate mean sphere, cylinder, and axis, M, J0, and J45 differences by cycloplegic drops according to the type of astigmatism, patients were divided into three groups according to the axis: with the rule (axis ≤ 30 and axis ≥ 150), oblique (30 < axis < 60), and against the rule (30 < axis < 60 and 120 < axis < 150). The data were analyzed by SPSS software version 16 (SPSS Inc., Chicago, IL). In order to investigate the significant changes in each of the variables following cycloplegia, a paired samples *t*-test was used. Mean sphere, cylinder, and axis, M, J0, and J45 differences by cycloplegic drops were compared between two groups by an independent sample *t*-test. The agreements between other refraction measurements with subjective refraction as the gold-standard procedure were evaluated using intraclass-correlation coefficient (ICC) for J0, J45, M, cylindrical power, and cylindrical axis.

## 3. Results

A total of 1014 eyes were included in this study. The mean age of the patients was 29.64 ± 5.84 years (range: 17 to 45 years). The patients were classified according to cylindrical power into two groups: lower astigmatism group (−2.25 to −0.50) and higher astigmatic group (−2.50 to over). The mean age in the lower astigmatism group (29.58; 95% CI: 29.18 to 29.99 years) was not significantly different from the higher astigmatic group (29.85; 95% CI: 29.07 to 30.62) and there were no significant differences in gender between these two groups (*P*=0.54). Two different methods were used to find the effect of cycloplegia on the power and axis of astigmatism. By using the first method, we calculated arithmetic changes of the refraction in sphere, cylindrical power, and axis under the influence of cyclopentolate, and the significant changes analyzed by paired *t*-test in both groups (Table 1). There were significant differences between post- and predrop for spherical and cylindrical power in the two groups (*P*=0.05). The sphere, cylindrical power, and axis differences by cycloplegic drop were significantly different between the two groups (Table 1). By the second method of analysis using Alpins method, differences between wet and dry refraction in J0 (−0.03; 95% CI:−0.06 to−0.008) and J45 (−.03; 95% CI:−0.06 to −0.01) were significant only in the higher astigmatic group. Axis changes by the cycloplegic drop in lower astigmatism group were 3.51 (CI: 3.22 to 3.81) and axis changes by the cycloplegic drop in higher astigmatism group was 2.21 (CI: 1.73 to 2.49). We found significant differences in axis changes by cycloplegic drop between two groups (*P* < 0.001). Based on the type of astigmatism, the individuals were divided into three groups: with the rule (724 eyes), against the rule (122 eyes), and oblique astigmatism (168 eyes). The shifts of sphere, cylinder, and axis, M, J0, and J45 by cycloplegia were shown in a different type of astigmatism (Table 2). Excellent ICC values were found between subjective refraction and cyclorefraction (Table 3). In the high astigmatic group, ICC coefficients between cylindrical components of subjective refraction and autorefraction were a little more than these values between subjective refraction and cyclorefraction (Table 3).

## 4. Discussion

High astigmatism showed the greatest variation in accommodation [18]. There is a concern about the possibility of changing the cylindrical power and axis by accommodation. In the refraction of astigmatism, precise determination of power and axis for distance and near is essential. In routine clinical practice, it has often been seen that astigmatic patients require separate prescription for near-work activity due to visual symptoms [16, 17, 20]. In our study, power vectors J0, J45 and, M changing by cycloplegia were significantly different between low and high astigmatic power groups (*P*=0.016) (*P*=0.024) (*P*=0.003). Mean of axis changes by cycloplegia in group 1 (astigmatism power −.50 to −2.250D) was significantly more than group 2 (astigmatism power < −2.25) (*P* < 0.001). J0 and J45 changing by cycloplegia in group by high astigmatism (group 2) were more negative than group 1. This means that astigmatism power could be more negative by cycloplegic. On the other hand, sphere and M values by cycloplegia are more positive than these values by dry refraction. One of the most important factors in refraction is the control of the accommodation. If the patient has astigmatism, there is a concern about the possibility of changing the cylindrical power and axis by accommodation [12]. We compared the cylindrical power before and after cycloplegia in these two groups (Table 1). The cylindrical powers before and after cycloplegia were not significantly different. This is probably due to the simultaneous analysis of different axis of astigmatism. This result is in line with previous studies [11, 13]. The Alpins method was used and the shifts of M, J0, and J45 by cycloplegia were analyzed. As shown in Table 1, the patients with higher cylindrical power (group 2) had more J0, J45, and M changing by cycloplegic than the patients with less astigmatic power (group 1). A similar pattern of results about J0 changes was obtained by Jorge et al. in 2005 [21], Radhakrishnan et al. in 2007 [22], and Asharlous et al. in 2016 [23]. These findings are consistent with Cheng et al.'s research in 2004 showing that coma and astigmatism were also changed with accommodation [24], but the direction of the change was not clear. It is possible that the mydriasis caused by the cycloplegic effect could lead to high order aberration growth [25, 26]. Wang concluded that coma-like aberration was larger than other higher-order aberrations for all pupil sizes [25]. This increase in coma aberration may be due to the effect of cycloplegia on accommodation because it can be seen with different pupil sizes [25]. After dividing all patients according to the type of astigmatism into three groups (WTR, ATR, and OBL), we found these mean J0 differences by cycloplegic effects were −0.02 ± 0.16 in the WTR astigmatism group and 0.03 ± 0.11 in the ATR astigmatism group. If we accept the cycloplegic effect as an accommodative relaxation, it was expected to have less ATR astigmatism (more negative in the minus cylinder) and more WTR astigmatism (less negative in the minus cylinder) by accommodation. It can be said that the patients exhibit with-the-rule astigmatism during accommodation, a study reported similar to this conclusion [27]. Wills et al. found that ATR astigmatism has significantly greater effects on reading performance than WTR astigmatism [8]. It can be argued that the accommodation would help to reduce the symptom of reading performance. On the other hand, the astigmatic accommodation hypothesis is again raised [22, 28]. According to this theory, total astigmatism compensation might occur through accommodation by sector contraction ciliary muscle, which could be compensatory or accidental. Our results show that there is a significant shift along the 180 and 90, but there was no significant shift along the 45 and 135. Other studies also reported that accommodation function leads to a change in WTR astigmatism since the lens starts tilting around the horizontal axis [22, 27]. Marginal astigmatism aberration can be created as a result of spherical lens tilting, which is called induced cylinder [29]. The tilting lens makes a difference between the dioptric power of the tangential and sagittal meridian of the spherical lens. Astigmatism axis variations were significantly different between two astigmatic groups (*P* < 0.001). The maximum axis change by cycloplegia was observed in the oblique astigmatism group (Table 2). Similar results were reported in previous case reports [16], stating that patients with distant oblique astigmatism required separate prescription for near-work activities due to the shift of cylindrical axis by accommodation. There were several cases of change in the power and axis of astigmatism with cycloplegic drops in clinical practice [18, 23]. There are several theories about axis change through accommodation. Four theories had been explained. The first was that the shift in the axis is caused by the lenticular change. The second was about fusional compensation which led to a slight shift in axis. The third theory was cyclophoria (torsional movements of the globe). The last one was about the change of corneal curvature because of the extrinsic muscles in near vision [20, 28]. We found the maximum mean axis change by cycloplegia in oblique astigmatism group. To understand the mechanism of accommodation, it could be said that horizontal and vertical zonular changes are more than oblique zonular changes and astigmatic accommodation could be supported. If the patient has refractive error of astigmatism at far, accurate correction of the axis and power of astigmatism is necessary for near-work activity by subjective test. Among subjective refraction, it could be consider that the patients with the rule and against the rule astigmatism are more likely to change power and the patients by oblique astigmatism are more likely to change axis for near-work activities and accommodation. We did not observe statistically significant changes in J0 and J45 by cycloplegia in group 1, but significant differences were found in J0 and J45 by cycloplegia in group 2. It could be said that J0 and J45 changing by cycloplegic were more in patients with high astigmatic power. In estimation of the agreement between dry autorefractor, cycloplegic autorefractor with the subjective refraction, it can be seen from data that ICC coefficients for sphere, cylindrical power, M, J0, and J45 between dry and cyclorefraction with subjective refraction were excellent in both groups. A limitation of our study is that the groups of three types of astigmatism were not matched according to age and cylindrical power so it is not possible to have comparison between them. Another limitation is to try to predict near-work problems based on the results in patients' distant vision and cycloplegic results.

## 5. Conclusions

In conclusion, during accurate refractive measurements, it is suggested that an additional near subjective refraction was done in patients with a lower amount of astigmatism (−2.25 to −0.50) for determining the axis of astigmatism. Moreover, an additional near subjective refraction might be done for precise determination of astigmatic power in patients with a higher amount of astigmatism (−2.50 to over). So, it could help us to anticipate which astigmatic patients need a separate prescription for near-work activities and where it is more likely that this difference between near and far astigmatic values would occur.

## Figures and Tables

**Table 1:** Comparison of mean differences of sphere, power, axis, M, J0, and J45 before and after cycloplegia in different cylindrical power groups.

	Lower astigmatic power	Higher astigmatic power	*P* value
Sphere	0.22 ± 0.46	0.33 ± 0.70	0.035
	*P* < 0.0001	*P* < 0.0001	
Cylinder	0.03 ± 0.25	0.07 ± 0.46	0.209
	*P* < 0.0001	*P*=0.012	
Axis	3.51 ± 4.23	2.11 ± 2.86	<0.0001
	Range (0–30)	Range (0–20)	
M	0.24 ± 0.47	0.37 ± 0.73	0.016
	*P* < 0.0001	*P* < 0.0001	
J0	−0.002 ± 0.13	−0.03 ± 0.22	0.024
	*P*=0.593	*P*=0.012	
J45	0.003 ± 0.09	−0.03 ± 0.24	0.003
	*P*=0.315	*P*=0.015	

Lower astigmatic power = −2.25 to −0.50, higher astigmatic power = −2.50 to over.

**Table 2:** Changes of the refraction in sphere, power, and axis, M, J0, and J45 before and after cycloplegia in different types of astigmatism.

Value	WTR	OBL	ATR
Sphere	0.25 ± 0.51	0.26 ± 0.63	0.24 ± 0.47
Cylinder	0.03 ± 0.33	0.08 ± 0.28	0.05 ± 0.24
Axis	2.89 ± 3.69	4.29 ± 5.17	3.59 ± 3.78
J0	−0.02 ± 0.16	0.01 ± 0.13	0.03 ± 0.11
J45	−0.009 ± 0.13	0.001 ± 0.14	0.01 ± 0.10
M	0.26 ± 0.53	0.30 ± 0.64	0.27 ± 0.48

WTR = with the rule astigmatism, OBL = oblique astigmatism, and ATR = against the rule astigmatism.

**Table 3:** Intraclass-correlation coefficient between subjective refraction for sphere, cylinder, axis, M, J0, and J45.

		Lower astigmatic power	Higher astigmatic power
Sphere	Subdry	ICC = 0.927	ICC = 0.930
		*P* < 0.0001	*P* < 0.0001
	Subwet	ICC = 0.932	ICC = 0.935
		*P* < 0.0001	*P* < 0.0001
Cylinder	Subdry	ICC = 0.886	ICC = 0.917
		*P* < 0.0001	*P*=0.0001
	Subwet	ICC = 0.90	ICC = 0.886
		*P* < 0.0001	*P* < 0.0001
Axis	Subdry	ICC = 0770	ICC = 0.745
		*P* < 0.0001	*P* < 0.0001
	Subwet	ICC = 0.775	ICC = 0.746
		*P* < 0.0001	*P* < 0.0001
M	Subdry	ICC = 0.926	ICC = 0.924
		*P* < 0.0001	*P* < 0.0001
	Subwet	ICC = 0.931	ICC = 0.931
		*P* < 0.0001	*P* < 0.0001
J0	Subdry	ICC = 0.961	ICC = 0.962
		*P* < 0.0001	*P* < 0.0001
	Subwet	ICC = 0.965	ICC = 0.948
		*P* < 0.0001	*P* < 0.0001
J45	Subdry	ICC = 0.959	ICC = 0.954
		*P* < 0.0001	*P* < 0.0001
	Subwet	ICC = 0.963	ICC = 0.936
		*P* < 0.0001	*P* < 0.0001

Lower astigmatic power = −2.25 to −0.50, higher astigmatic power = −2.50 to over.

## Data Availability

Data are available upon reasonable request.
